# Anthocyanins attenuate endothelial dysfunction through regulation of uncoupling of nitric oxide synthase in aged rats

**DOI:** 10.1111/acel.13279

**Published:** 2020-12-03

**Authors:** Geum‐Hwa Lee, The‐Hiep Hoang, Eun‐Soo Jung, Su‐Jin Jung, Seong‐Kyu Han, Myoung‐Ja Chung, Soo‐Wan Chae, Han‐Jung Chae

**Affiliations:** ^1^ Non‐Clinical Evaluation Center Biomedical Research Institute Jeonbuk National University Hospital Jeonju Korea; ^2^ Clinical Trial Center for Functional Foods (CTCF2) Jeonbuk National University Hospital Jeonju Korea; ^3^ Department of Oral Physiology School of Dentistry & Institute of Oral Bioscience Jeonbuk National University Jeonju Korea; ^4^ Department of Pathology Jeonbuk National University Medical School Jeonju Korea; ^5^ School of Pharmacy Jeonbuk National University Jeonju Korea; ^6^ Research Institute of Clinical Medicine of Jeonbuk National University‐Biomedical Research Institute of Jeonbuk National University Hospital Jeonju Korea

**Keywords:** anthocyanins, eNOS deacetylation, NO, senescence, SIRT1

## Abstract

Endothelial dysfunction is one of the main age‐related arterial phenotypes responsible for cardiovascular disease (CVD) in older adults. This endothelial dysfunction results from decreased bioavailability of nitric oxide (NO) arising downstream of endothelial oxidative stress. In this study, we investigated the protective effect of anthocyanins and the underlying mechanism in rat thoracic aorta and human vascular endothelial cells in aging models. In vitro, cyanidin‐3‐rutinoside (C‐3‐R) and cyanidin‐3‐glucoside (C‐3‐G) inhibited the d‐galactose (d‐gal)‐induced senescence in human endothelial cells, as indicated by reduced senescence‐associated‐β‐galactosidase activity, p21, and p16^INK4a^. Anthocyanins blocked d‐gal‐induced reactive oxygen species (ROS) formation and NADPH oxidase activity. Anthocyanins reversed d‐gal‐mediated inhibition of endothelial nitric oxide synthase (eNOS) serine phosphorylation and SIRT1 expression, recovering NO level in endothelial cells. Also, SIRT1‐mediated eNOS deacetylation was shown to be involved in anthocyanin‐enhanced eNOS activity. In vivo, anthocyanin‐rich mulberry extract was administered to aging rats for 8 weeks. In vivo, mulberry extract alleviated endothelial senescence and oxidative stress in the aorta of aging rats. Consistently, mulberry extract also raised serum NO levels, increased phosphorylation of eNOS, increased SIRT1 expression, and reduced nitrotyrosine in aortas. The eNOS acetylation was higher in the aging group and was restored by mulberry extract treatment. Similarly, SIRT1 level associated with eNOS decreased in the aging group and was restored in aging plus mulberry group. These findings indicate that anthocyanins protect against endothelial senescence through enhanced NO bioavailability by regulating ROS formation and reducing eNOS uncoupling.

## Graphical abstract

Aging is known for its correlation with increased protein acetylation rates and the decline of sirtuin‐1 (SIRT1) deacetylation activity. This study demonstrated that anthocyanin‐rich mulberry extract reduces oxidative stress in aging vasculature and attenuates endothelial dysfunction through reversed inhibition of endothelial nitric oxide synthase (eNOS), serine phosphorylation, and SIRT1 expression, recovering NO level in senescence.

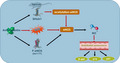

## 1 INTRODUCTION

The life expectancy of older adults is increasing rapidly due to advancements in medical and health care. However, age‐related decline in health has not received adequate attention, presenting many problems to the healthcare system (Newgard & Sharpless, 2013). The endothelial dysfunction is one of the main age‐related arterial phenotypes known to be responsible for cardiovascular disease (CVD) in older adults. Besides, endothelial dysfunction is modulated by mainstream CVD risk factors in older adults in addition to reduced bioavailability of nitric oxide (NO), downstream of endothelial oxidative stress, and inflammation (Donato et al., 2015).

Oxidative stress may be characterized as a state in which reactive oxygen species (ROS) bioavailability is increased with decreasing antioxidant defenses (Price et al., 2000). In aging, greater endothelial oxidative stress is a result of increased production of uncoupled endothelial nitric oxide synthase (eNOS) and intracellular enzyme nicotinamide adenine dinucleotide phosphate hydrogen (NADPH) oxidase as well as from mitochondrial respiration, leading to endothelial dysfunction (Donato et al., 2015). Endogenous NO produced from eNOS plays a key role in maintaining endothelial function homeostasis and regulation of blood vessel tone, leukocyte adhesion, proliferation, and migration of smooth muscle cells and platelet aggregation (Yumeng & Yu, 2018). The eNOS also contributes to oxidative stress resistance by generating NO and inhibiting O_2_^−^ generation (Forstermann & Sessa, 2012). Several novel cellular signaling pathways tend to modulate aged endothelial activity. The eNOS is under significant regulatory control. The enzyme undergoes acetylation, nitrosylation, and phosphorylation that either positively or negatively control NO synthetic potential depending on the modification or site (Alderton et al., 2001).

Aging is known to correlate with higher rates of protein acetylation and the decline of sirtuin 1 (SIRT1) deacetylation activity, which may partly underlie the diminished eNOS activity (Donato et al., 2011; Kitada et al., 2016). SIRT1 belongs to the sirtuin family of enzymes related to lifespan extension as well as other anti‐aging effects (Mitchell et al., 2014). The SIRT1 role relates to deacetylation and thus modulates the activity of nuclear transcription factors, co‐regulators, and proteins to adjust gene expression in response to the cellular energy condition and provide “stress resistance” by pro‐inflammatory and oxidative stress pathways (Yeung et al., 2004). SIRT1 affects the regulation of transcription and enzymatic activity of eNOS in the endothelium, leading to an increase in NO production (Xia et al., 2013). SIRT1 also enhances the enzymatic activity of eNOS by deacetylation (Man et al., 2019). SIRT1 was reported to modulate NO and endothelial function directly through deacetylation and subsequent activation of eNOS in small and large arteries (Chang & Guarente, 2014). Phenolic compounds, flavonoids, and anthocyanins, which are active substances in vegetables and fruits, have potent antioxidant effects and are now considered for developing a potential strategy for the prevention of endothelial dysfunction and cardiovascular pathological conditions (Furuuchi et al., 2018). Mulberry is rich in phenols, flavonoids, anthocyanins, and many other antioxidants, and has been used in foods and pharmaceuticals because of its pharmacological effects. Previously, the effects of cyanidin‐3‐rutinoside (C‐3‐R) and cyanidin‐3‐O‐β‐glucoside (C‐3‐G), the most abundant anthocyanins in mulberry, have attenuated endothelial dysfunction in high‐fat diet model. Thus, the present research was undertaken to investigate the impact of anthocyanins and the underlying mechanisms and effect of the anthocyanin‐rich mulberry extract on endothelial senescence and aging rats.

## 2 RESULTS

### 2.1 Anthocyanin‐rich mulberry extract alleviated in vivo endothelial senescence and oxidative stress in the aorta of aging rats

**Figure 1:**
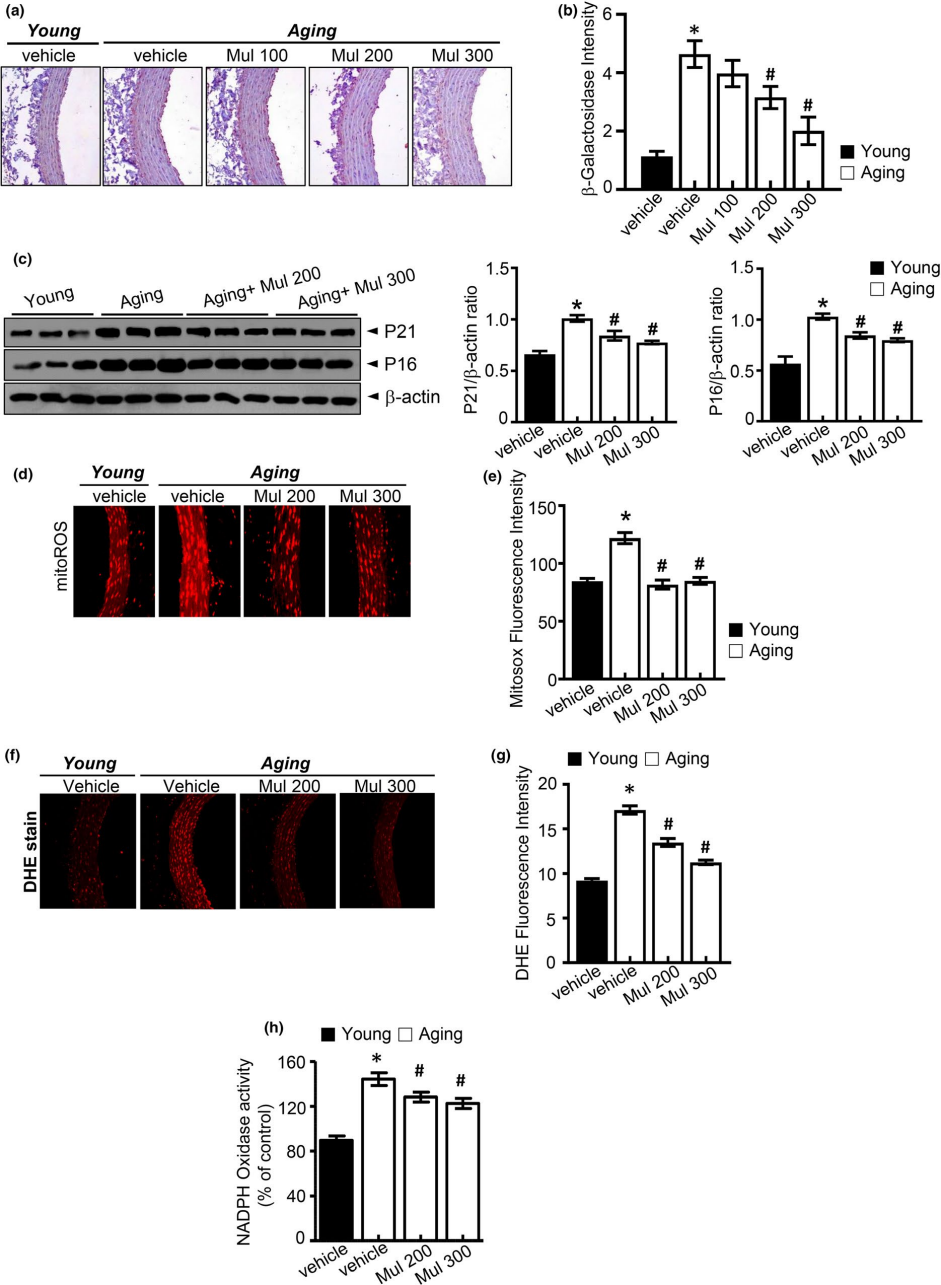
Anthocyanin‐rich mulberry extract inhibits aging‐induced senescence and oxidative stress in aging rats. (a, b) SA‐β‐gal staining of the aortas. Quantification analysis of SA‐β‐gal expression. (c) p21, p16, and expression of β‐actin in the aortas were determined by Western blotting. (d) Representative images of MitoSOX staining for MitoROS production and (e) quantification of fluorescence intensity for superoxide levels in aortas. (f) Representative fluorescent images of ROS formation in aortas were shown using ROS probe DHE. (g) Quantification of fluorescence intensity for superoxide levels in aortas. (h) NADPH oxidase activity determined by lucigenin chemiluminescence assay. Values are presented as mean ± SEM (*n* = 6, *p < 0.05 vs. the young group; #*p* < 0.05 vs. the aging group)

Eighty‐week‐old rats received 8 weeks of treatment of anthocyanin‐rich mulberry extract to observe the in vivo effect. Eight‐week‐old rats were served as the young group, while the untreated aging rats were served as the model group. Body weights were unaltered in mulberry‐administered aging rats compared with aged rats (Figure S1). As shown in Figure 1a,b, the aortas of aging rats had significantly higher SA‐β‐gal staining levels compared to the young group. In aortas of mulberry‐treated aging group, a significant decrease in SA‐β‐gal staining signal was observed as compared to that of the untreated aging group. Also, while aortic wall thickness was significantly increased in the aging group compared to the young group. In contrast, aortic wall thickness was not significantly reduced in aging plus mulberry group compared with aging group (Figure S2). Additionally, mulberry extract treatment decreased the senescence markers p16 and p21 in the aging plus mulberry group (Figure 1c). These results confirm that mulberry extract prevents endothelial senescence. We examined the effect of orally administered mulberry extract on oxidative stress in aging rats. There was a significant increase in MitoSOX fluorescence throughout the aortic wall of aging rats compared to young rats but was reversed by mulberry extract supplement (Figure 1d,e). Expectedly, mulberry extract controlled the highly stimulated DHE fluorescence in aorta rings from the aging condition (Figure 1f,g). In the cell, NADPH oxidase system is a significant producer of ROS and plays a crucial role in intracellular ROS homeostasis (Dröge, 2002). In Figure 1h, NADPH oxidase activity was significantly increased in the aging group compared to the young group but was reversed by mulberry extract supplement. Collectively, these results indicate that anthocyanin‐rich mulberry extract inhibits oxidative stress and redox imbalance in vascular senescence.

### 2.2 Anthocyanin‐rich mulberry extract increased production of NO in aging rats

**Figure 2:**
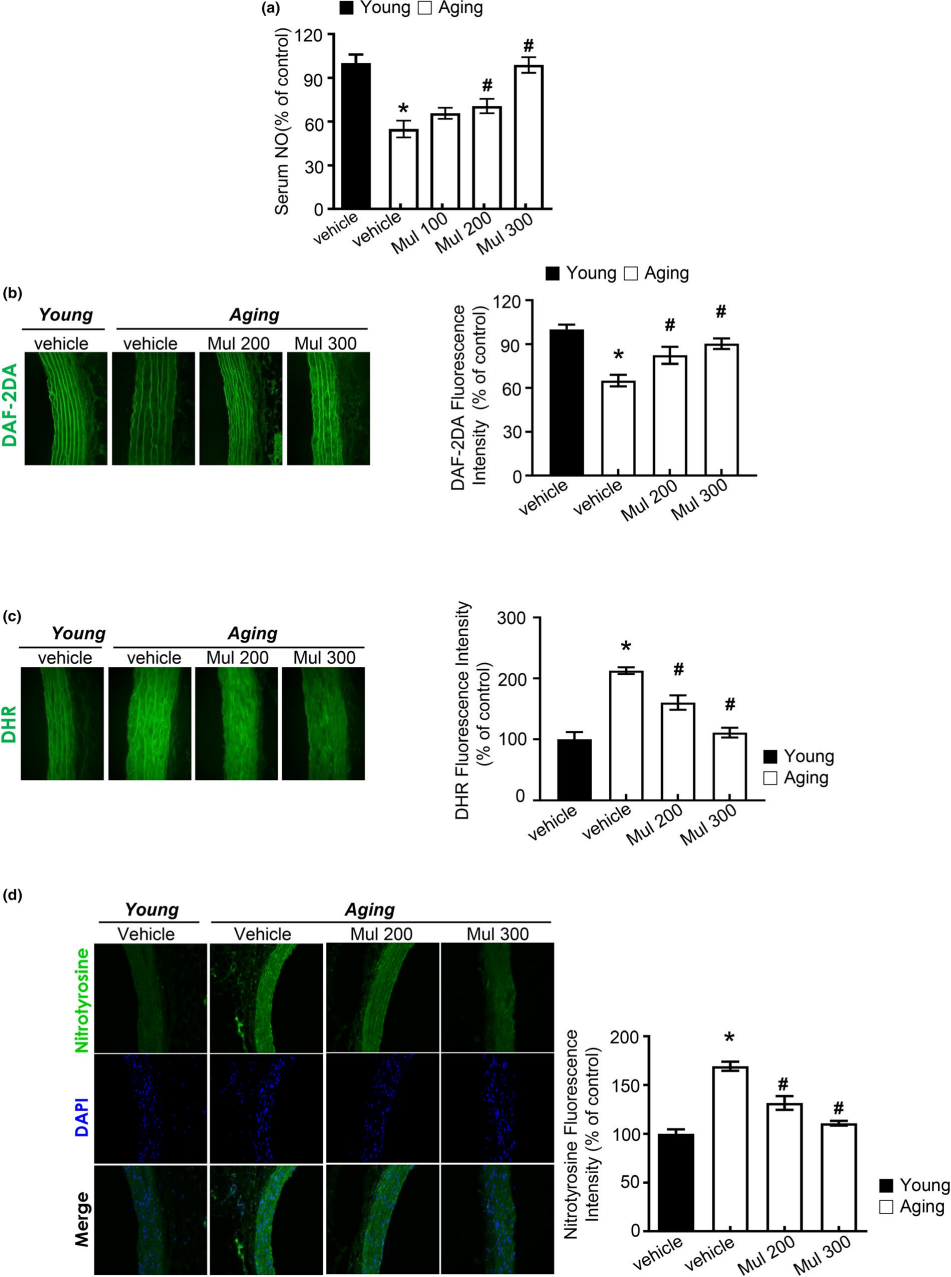
Anthocyanin‐rich mulberry extract improves vascular endothelial function in aging rats. (a) Level of NO was measured in rat serum of different experimental groups. Relative quantification of NO in serum. (b) DAF2DA fluorescence staining for NO levels in aortas. (c) Representative images of DHR staining for peroxynitrite in aortas and quantification of DHR fluorescence intensity for peroxynitrite levels in aortas. (d) Nitrotyrosine expression in the aortas was determined by immunofluorescence (*n* = 6, *p < 0.05 vs. the young group; #*p* < 0.05 vs. the aging group)

Endothelium‐derived NO is known to be particularly important for maintaining normal vascular function. The activity of eNOS and the production of NO are diminished in senescent human endothelial cells (Hayashi et al., 2008). Several studies have reported the impairment of eNOS function and decrease of NO production in the progression of lipid dysmetabolic state. Triglyceride (TG), total cholesterol (TC), and LDL‐c (LDL‐cholesterol) levels in serum were significantly increased in the aging group compared to the control group. After 8 weeks of treatment, serum levels of TG, TC, and LDL were significantly reduced in both the aging plus mulberry group compared to the aging group (Figure S3a). The fasting plasma glucose concentrations were unaltered in mulberry‐administered aging rat compared with aged rat (Figure S3b). As shown in Figure 2a,b, NO in serum and the fluorescence intensity of NO in aortas were decreased in aging group but were recovered by mulberry extract treatment. In addition, mulberry extract markedly attenuated peroxynitrite (Figure 2c) and reduced levels of nitrotyrosine (Figure 2d) in aortas. These data suggest that aging‐related abnormal superoxide anion accumulation is suggestive to reduce NO bioavailability and convert NO into peroxynitrite, one of the endothelial dysfunction mechanisms, whereas mulberry extract controls the aging‐related NO disturbance.

### 2.3 Anthocyanin‐rich mulberry extract improves eNOS function in aging rats

**Figure 3:**
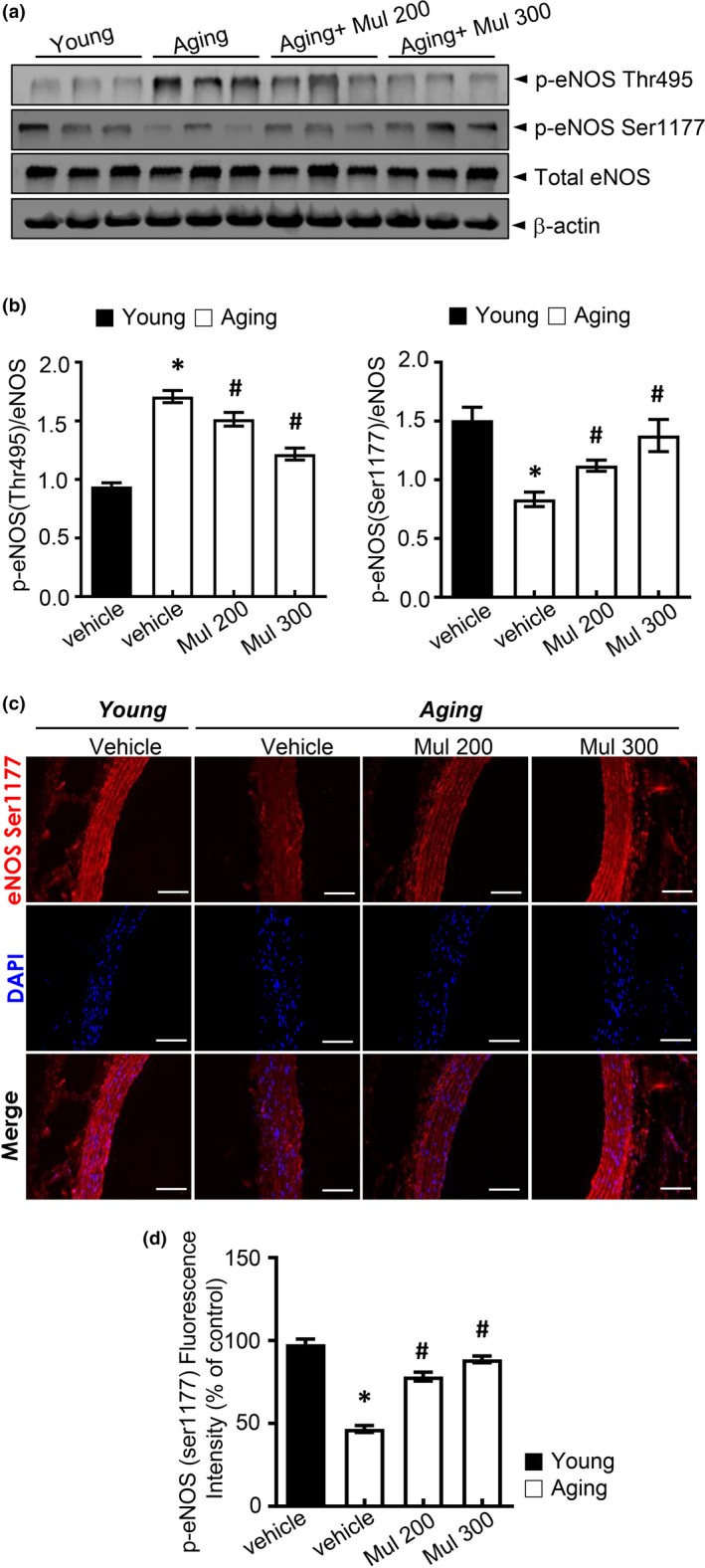
Mulberry extract improved eNOS activity in aging rats. (a, b) Phosphorylation of Ser1177 and Thr495 and expression of total eNOS and β‐actin in the aorta were determined by Western blotting. (c) Representative images of the immunofluorescence staining of phosphorylation of eNOS at Ser1177 (p‐eNOS) on aortic sections. (d) Quantification of phosphorylation of eNOS at Ser1177. Values are presented as mean ± SEM (*n* = 6, *p < 0.05 vs. the young group; #*p* < 0.05 vs. the aging group)

We examined the activity of eNOS in aging rats to illustrate the anti‐aging effects of mulberry in vivo. The eNOS is dynamically regulated by changes in protein phosphorylation. Mulberry extract supplement at the aortic rings decreased the phosphorylation of eNOS on Thr495 and increased the phosphorylation of eNOS Ser‐1177, compared to the aging group (Figure 3a,b). Consistently, mulberry extract recovered eNOS on Ser‐1177 phosphorylation in the aorta of aging rats (Figure 3c,d), showing the role of mulberry extract on the eNOS activity through the regulation of eNOS phosphorylation in aging.

### 2.4 Anthocyanin‐rich mulberry extract improves acetyl‐lysine eNOS‐SIRT1 function in aging rats

**Figure 4:**
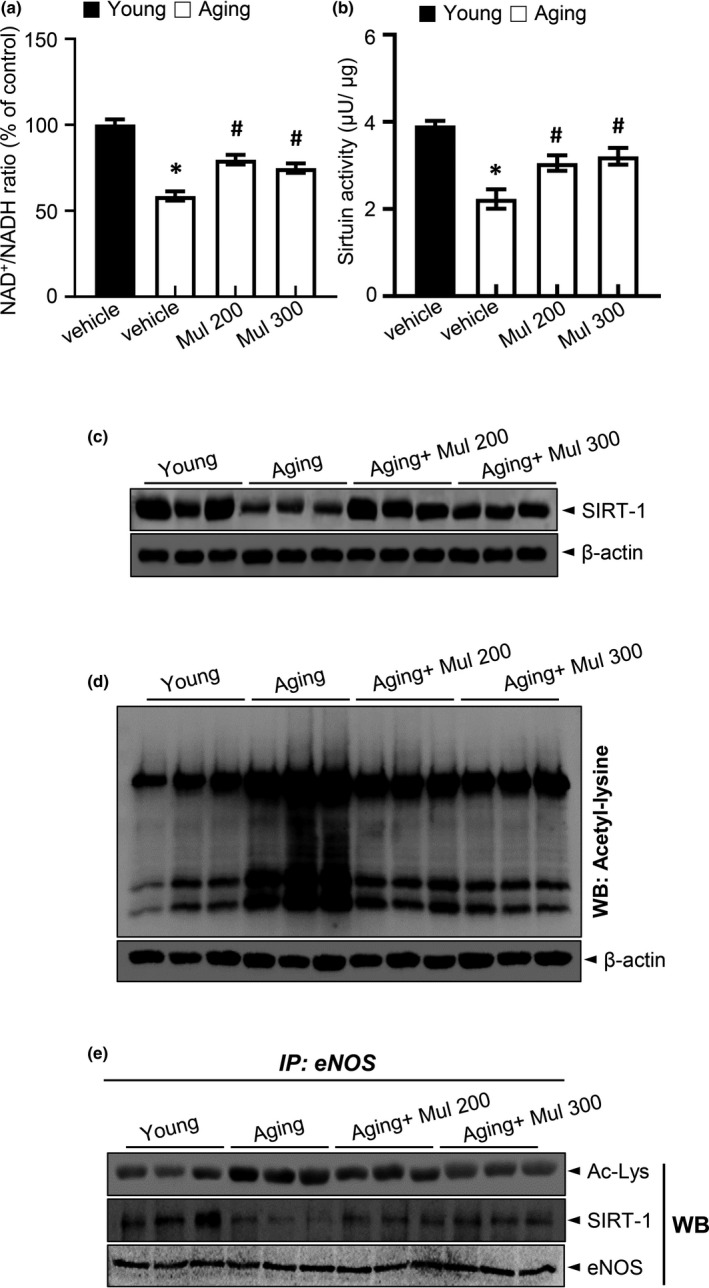
Mulberry extract improved eNOS activity in aging rats. (a) NAD^+^ and NADH levels were measured in the aortas, and NAD^+^/NADH ratios were quantified. (b) Sirtuin activity in the aortas. (c) SIRT1 and expression of β‐actin in the aortas were determined by Western blotting. (d) Acetylated lysine and expression of β‐actin in the aortas were determined by Western blotting. (e) Immunoprecipitation using an antibody against eNOS to evaluate acetylation levels and its association with SIRT1. Values are presented as mean ± SEM (*n* = 6, *p < 0.05 vs. the young group; ^#^ *p* < 0.05 vs. the aging group)

SIRT1 increases eNOS enzymatic activity by deacetylation. In endothelium, SIRT1 is physically associated with eNOS. Knock‐down of SIRT1 or inhibition of SIRT1 activity enhances eNOS acetylation on lysine 496 and 506 residues in the calmodulin‐binding domain (Mattagajasingh et al., 2007). In this study, mean levels of aortic NAD^+^/NADH ratio, sirtuin activity, and SIRT1 expression were lower in the aging control group when compared with the young control group. Mulberry extract supplementation recovered NAD^+^/NADH ratio, sirtuin activity, and SIRT1 expression in aging plus mulberry group (Figure 4a,c). The total protein acetyl‐lysine level was significantly increased in the aging group but was recovered by mulberry extract supplement (Figure 4d). Figure 4e shows eNOS acetyl‐lysine levels as well as the levels of SIRT1 associated with eNOS following immunoprecipitation. The eNOS acetylation was higher in the aging group and was restored by mulberry extract treatment. SIRT1 levels associated with eNOS were decreased in the aging group and were restored in aging plus mulberry group.

### 2.5 Anthocyanin and anthocyanin‐rich mulberry extract attenuate d‐gal‐induced senescence and oxidative stress in HUVECs

**Figure 5:**
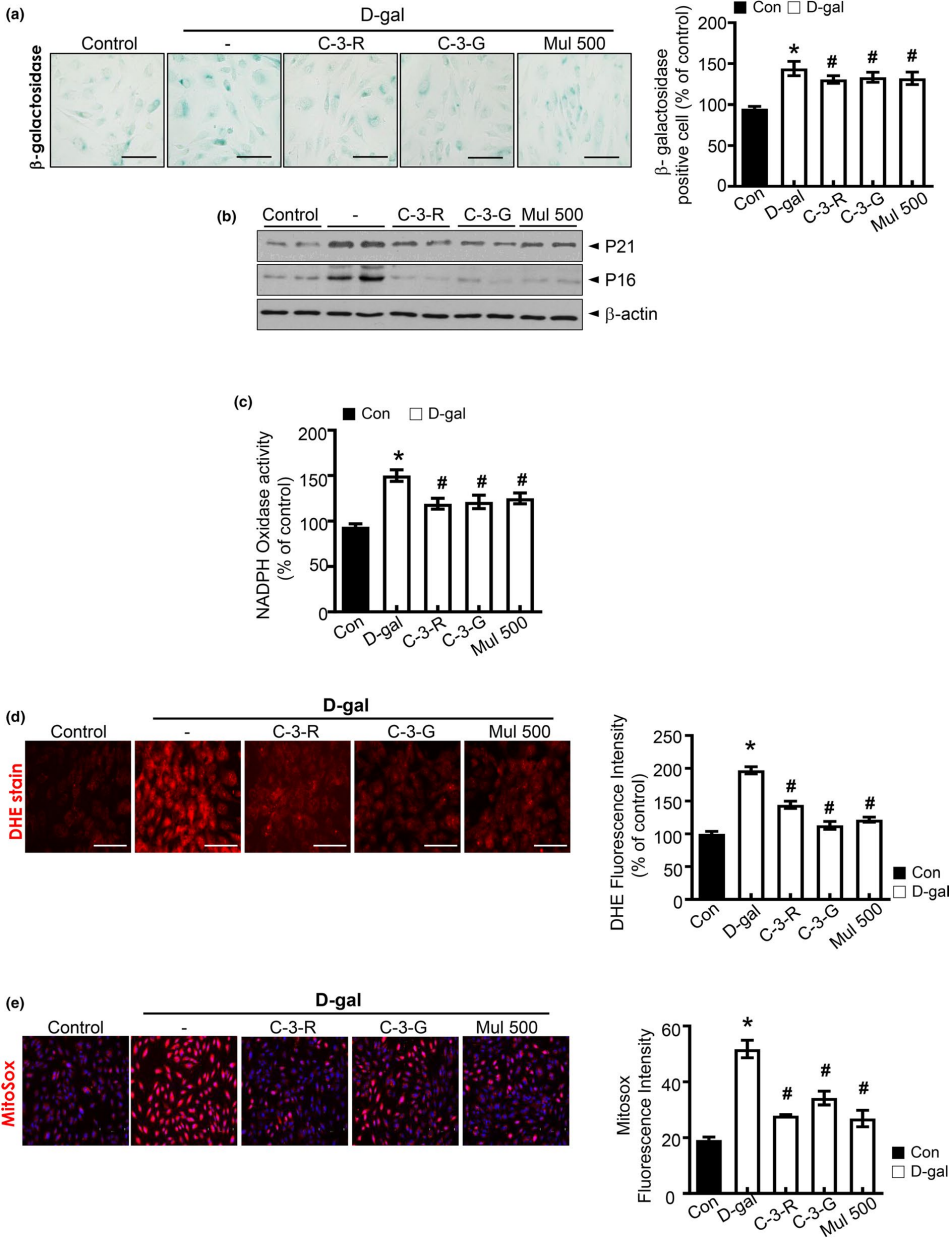
Anthocyanins inhibit d‐gal‐induced endothelial senescence and oxidative stress in HUVECs. (a) Representative images of senescence‐associated SA‐β‐gal staining in HUVECs are shown. Quantification analysis of positive SA‐β‐gal staining cell number from three biological replicates. (b) Western blot analysis of the level of p16 and p21. Equal protein loading was verified by measurement of β‐actin level. (c) NADPH oxidase activity determination by lucigenin chemiluminescence assay. (d) Representative fluorescent images of ROS formation in HUVECs were shown using ROS probe DHE. HUVECs were pre‐incubated with C‐3‐R, C‐3‐G, or mulberry extract for 30 min prior to d‐gal treatment. Quantification analysis of ROS production. (e) Representative images of MitoSOX staining for MitoROS production and quantification of fluorescence intensity for superoxide levels in HUVECs. Values are expressed as mean ± SE (*n* = 5, ^*^ *p* < 0.05 vs. control; ^#^ *p* < 0.05 vs. d‐galactose group)

To identify the effect of anthocyanin on cellular senescence in cultured HUVECs, we evaluated senescence markers, including SA‐β‐gal activity, p16^INK4a^, and p21 protein expression. Representative images showed that d‐gal treatment induced cellular senescence in HUVECs, which is characterized by increased activity of senescent marker SA‐β‐gal compared with the control group, but exposure to mulberry extracts containing C‐3‐R, C‐3‐G, and its metabolites reversed the effect (Figure 5a). Quantitative analysis of positive SA‐β‐gal staining cells confirmed the results. Senescent markers p16^INK4A^, p21 were also evaluated. C‐3‐R, C‐3‐G, and mulberry extract blocked d‐gal‐induced p16^INK4A^ and p21 expression (Figure 5b). Therefore, C‐3‐R, C‐3‐G, and mulberry extract are capable of blocking d‐gal‐induced endothelial senescence. Next, we assessed the effect of anthocyanin and mulberry extract on d‐gal‐induced oxidative stress. Representative images showed that C‐3‐R, C‐3‐G, and mulberry extract attenuated d‐gal‐induced ROS formation in human endothelial cells, which was verified by quantitative analysis. Similarly, C‐3‐R, C‐3‐G, and mulberry extract antagonized d‐gal‐induced NADPH oxidase activity (Figure 5c). To further determine the anthocyanin effect on senescence‐associated oxidative stress in HUVECs, fluorescence probe DHE was used to detect cellular ROS formation. Representative images show that C‐3R, C‐3‐G, and mulberry inhibited d‐gal‐induced accumulation of cellular ROS in HUVECs (Figure 5d). There was a significant increase in MitoSOX fluorescence throughout the d‐gal‐induced senescent HUVECs compared to the control but was reversed by C‐3‐R, C‐3‐G, and mulberry extract (Figure 5e). These results indicate that C‐3‐R, C‐3‐G, and mulberry inhibit d‐gal‐induced oxidative stress in endothelial cells.

### 2.6 Anthocyanin increased the production of NO in senescent HUVECs

**Figure 6:**
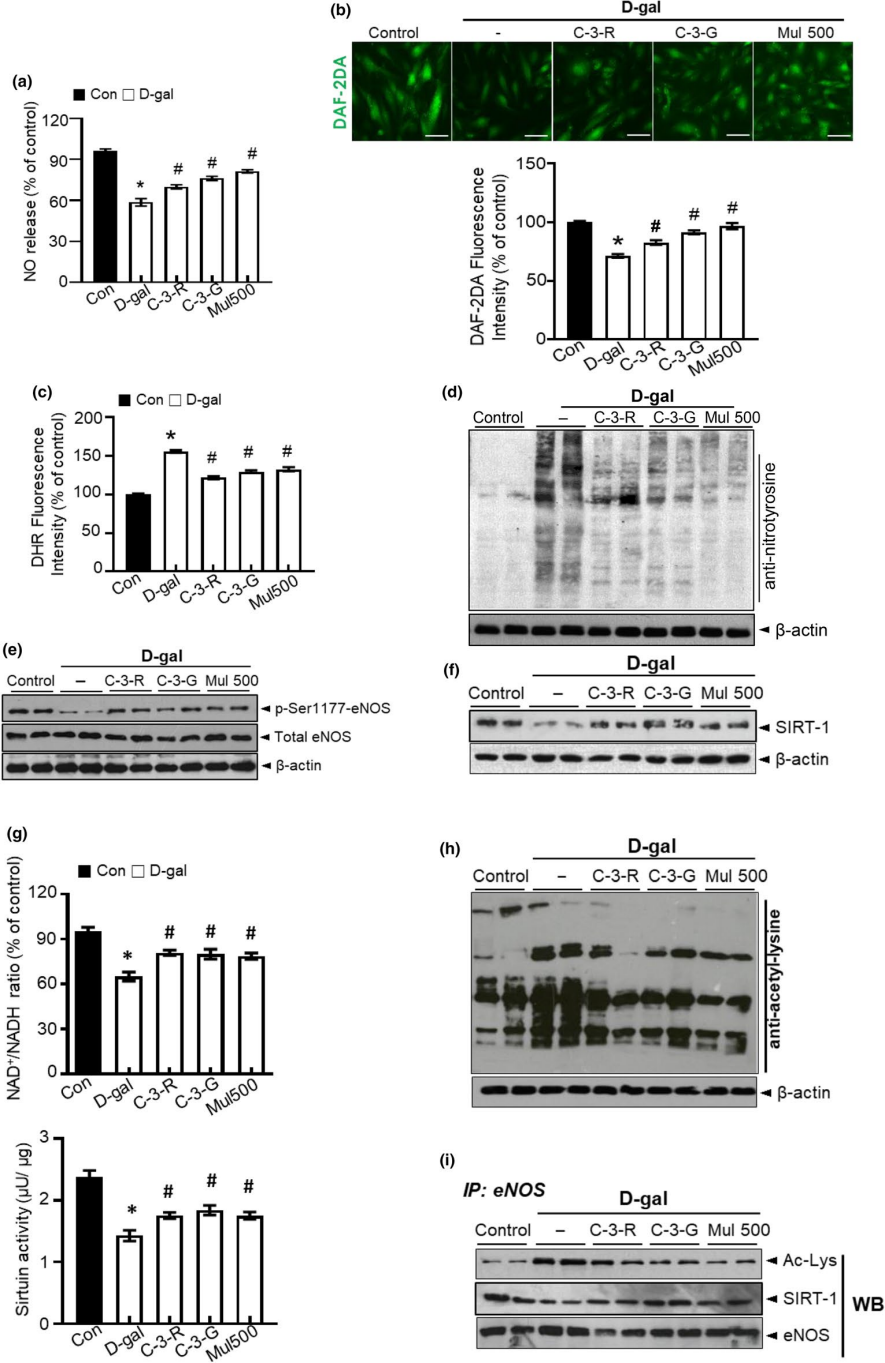
Anthocyanin regulates the effects of d‐gal on NO production and eNOS acetylation. HUVECs were treated with C‐3‐R, C‐3‐G, and mulberry extract, and then incubated with d‐gal for another 48 h. (a) NO release was measured as described in Materials and Methods. (b) Intracellular NO formation was measured via image analysis of NO‐sensitive fluorochrome, DAF‐2DA. (c) Quantification of DHR fluorescence intensity for peroxynitrite levels. (d) Nitrotyrosine and expression of β‐actin in HUVECs were determined by Western blotting. (e) Phosphorylation of eNOS at Ser1177 and expression of total eNOS and β‐actin in the HUVECs were determined by Western blotting. (f) SIRT1 and expression of β‐actin were determined by Western blotting. (g) NAD^+^ and NADH levels were measured, and NAD^+^/NADH ratios were quantified. Sirtuin activity. (h) Acetylated lysine and expression of β‐actin were determined by Western blotting. (i) eNOS was immunoprecipitated from HUVECs lysate, and its level of acetylation was analyzed by immunoblotting with anti‐acetyl‐lysine and anti‐SIRT1 antibody. Values are expressed as mean ± SE (*n* = 5, ^*^ *p* < 0.05 vs. control; ^#^ *p* < 0.05 vs. d‐galactose group)

Impairment of NO production is a hallmark of endothelial aging (Lee et al., 2010). Abnormalities in NO production by the vascular endothelium result in endothelial dysfunction (Minamino et al., 2002). In the present study, we measured NO, using DAF‐2DA probe to detect endothelial NO production (Balcerczyk et al., 2005). D‐galactose decreased NO production, but this effect was reversed by anthocyanin and mulberry extract (Figure 6a). As shown in Figure 6b, the fluorescence intensity of NO was reduced by d‐galactose treatment, but was reversed by C‐3‐R, C‐3‐G, and mulberry treatment. The fluorescent probe dihydroxy‐rhodamine (DHR) reacts with peroxynitrite‐derived free radicals, but not with O_2_^−^ or NO directly (Wrona et al., 2005). In addition, C‐3‐R, C‐3‐G, and mulberry extract markedly attenuated peroxynitrite and reduced levels of nitrotyrosine (Figure 6c,d) in HUVECs. We next examined eNOS phosphorylation status in the presence of C‐3‐R, C‐3‐G, and mulberry extract. D‐gal‐induced senescence significantly reduced the phosphorylation of Ser‐1177, while C‐3‐R, C‐3‐G, and mulberry treatment of HUVECs increased eNOS Ser1177 phosphorylation (Figure 6e). Aging‐related abnormal superoxide anion accumulation is suggestive to reduce NO bioavailability and to convert NO into peroxynitrite, one of the endothelial dysfunction mechanisms, whereas mulberry extract controls the aging‐related NO disturbance.

### 2.7 Anthocyanin improves acetyl‐lysine eNOS‐SIRT1 function in senescent HUVECs

SIRT1 is an important mediator in the progress of HUVECs senescence. We also found decreased SIRT1 activity and SIRT1 protein expression in d‐gal‐induced senescent HUVECs. C‐3‐R and C‐3‐G recovered SIRT1 protein expression and SIRT1 activity, NAD^+^/NADH ratio in d‐gal‐induced senescent HUVECs (Figure 6f,g). In senescent HUVECs induced by d‐gal with an increase in total protein acetyl‐lysine, which was essentially normalized using C‐3‐R, C‐3‐G, and mulberry extract treatment (Figure 6h). As shown, Figure 6i plots eNOS acetylation levels as well as levels of SIRT1 associated with eNOS following immunoprecipitation. The eNOS acetylation was higher in d‐gal‐treated cells and was restored by anthocyanin treatment. Sirtuin‐1 levels associated with eNOS were decreased in d‐gal‐treated cells and were restored by C‐3‐R, C‐3‐G, and mulberry extract treatment.

## 3 DISCUSSION

Aging is the main risk factor in developing chronic diseases, affecting multiple tissues of cardiovascular system, muscles, and bones. Age‐related diseases are the results of cellular damage accumulation and decreased activity of protective stress response pathways, leading to oxidative stress and low‐grade systemic inflammation (Serino & Salazar, 2018). Therapeutic strategies focusing on endothelial senescence have shed more light on cardiovascular disease treatment (Naylor et al., 2013).

D‐galactose induces accelerated aging mainly by increasing the oxidative stress level of the treated cells. High levels of d‐galactose can be converted into aldose and hydroperoxide under the catalysis of galactose oxidase, resulting in the generation of ROS. Increased ROS subsequently cause oxidative stress, inflammation, mitochondrial dysfunction, and apoptosis (Azman & Zakaria, 2019). In vitro, d‐galactose was used to induce HUVEC senescence. However, some limitations result in futile outcomes upon using d‐galactose‐induced aging model in vivo. In female reproductive aging studies, it was suggested that d‐galactose treatment is more suitable to induce polycystic ovary syndrome (PCOS) rather than aging (Park & Choi, 2012). Also, treatment of d‐galactose to 12‐week‐old Wistar rats reduced sperm motility index, but sexual incentive motivation, working memory, or object recognition were not affected (Tikhonova et al., 2014). Therefore, in our study, we used aging rats (80 weeks) for better representation of the aging physiology.

We have shown that aging is accompanied by the acceleration of oxidative stress in the vasculature and reduction in NO production, which are associated with decreased eNOS phosphorylation at activation sites and higher rates of amino acid acetylation. This research revealed that mulberry extract prevents both in vivo and in vitro endothelial dysfunction, providing evidence for its potential utility along with its main components in the age‐related endothelial dysfunction.

Mulberry extract is rich in phenolic components and flavonoids. The two main anthocyanins found in Korea's Rubus fruits are C‐3‐G and C‐3‐R (Jung et al., 2014). These anthocyanins are a subgroup of flavonoids, which have numerous physiological functions (Pandey & Rizvi, 2009). They have broad biological activities, including anti‐mutagenesis and anticarcinogenesis, which are generally attributed to their antioxidant activities.

Compared with the aging control group, the oxidative stress levels in aortas were downregulated in aging plus mulberry‐fed condition. Mulberry‐treated aortic rings of aging rats resulted in inhibition of the development of intracellular ROS, endothelial mitochondrial superoxide anion, and NADPH oxidase activity, suggesting that oxidative stress is intensified in the main subcellular organelles with extreme oxidative stress.

Since oxidative stress induces endothelial dysfunction primarily because superoxide rapidly inactivates NO and forms peroxynitrite, the formation of peroxynitrite may be another augmentation of signaling messenger to disrupt NO bioavailability. Expectedly, in the aging plus mulberry‐treated group, the ONOO^−^ regulation was observed compared to the aging control group. These abundant anthocyanins that finally regulated the release of NO seemed to correspond to the recovery of endothelial function through their antioxidant function. Indeed, in senescent endothelial cells, there is impairment of NO release and decrease of NO bioavailability (Lee et al., 2010). Treatment with DETA‐NO, a NO donor, or eNOS transfection protects against endothelial senescence (Zhong et al., 2010), while usage of eNOS inhibitor, such as L‐NAME, abrogates the anti‐senescent activity of NO and induces endothelial senescence (Boe et al., 2013). In this study, the mulberry extract treatment significantly improved NO production, which was detected by the DAF‐2DA fluorescent probe. The mechanisms underlying the NO regulation in aging endothelial are still undetermined. The two main tumor suppressors, p53 and p16/pRB, induce senescence (Campisi, 2005). Endothelial dysfunction is associated with telomere dysfunction in older adults and subsequently increases p53‐mediated cell cycle arrest through an increase in the expression of p21 cyclin‐dependent kinase inhibitor (Morgan et al., 2013). The p16/pRB pathway includes cyclin‐dependent kinase inhibitors 2A (p16)‐mediated inhibition of retinoblastoma‐like protein 1(pRB) (Stein et al., 1999). Observations confirm this conclusion that treatment with mulberry extract ameliorates aging expression of cell senescence marker SA‐β‐gal, downregulation of cell cycle inhibitor p21 and p16. This indicates that eNOS activation by mulberry extract is likely to be involved in its anti‐senescent properties. Since the activation of eNOS derives from the enzyme phosphorylation (Fleming & Busse, 2003; Förstermann & Münzel, 2006), the eNOS phosphorylation level was first determined to figure out how mulberry extract modulates activation of eNOS and NO formation. Serine 1177 and threonine 495 are the most important eNOS phosphorylation sites. The former increases Ca^2+^ sensitivity and represents an independent mechanism for activating eNOS, whereas the latter is a negative regulatory site, where phosphorylation of Thr495 can interact with binding of calmodulin at calmodulin‐binding site. This conclusion is supported by the finding that mulberry extract treatment reversed the reduced eNOS phosphorylation at Ser1177 caused by aging and enhanced eNOS activation and that mulberry extract did not alter the total expression of eNOS either. Recent evidence also points to a connection between the response to DNA damage and energy‐sensitive pathways, increasing activity, and expression of SIRT1. In the context of atherosclerosis, SIRT1 was shown to have a protective effect on human and rodent vascular cells against DNA damage (Gorenne et al., 2013). Conversely, reductions in the endothelial expression of SIRT1 with advancing age could lead to genomic instability. Knock‐down SIRT1 gene by siRNA prevents the eNOS upregulation by resveratrol in endothelial cells (Csiszar et al., 2009). Consistently, endothelium‐specific SIRT1 overexpression results in elevated expression of eNOS (Zhang et al., 2008). SIRT1 also increases the enzymatic activity of eNOS by deacetylation. In the endothelium, eNOS is physically related to SIRT1. Knock‐down or inhibiting activity of SIRT1 promotes eNOS acetylation in the calmodulin‐binding domain at lysine 496 and 506 residues (Mattagajasingh et al., 2007). Here, we focused on the ability of mulberry extract to restore levels of SIRT1 protein expression. Aging is associated with higher eNOS acetylation, an effect that has also been reversed by treatment with mulberry extract and related to improved protein–protein interaction of eNOS and SIRT1. C‐3‐G is an anthocyanin with antioxidant property and is shown to increase the expression of SIRT1 in a dose‐ and time‐dependent manner (Mogalli et al., 2018). Ota et al. reported that enhanced SIRT1 expression and physical coupling with eNOS improved endothelial senescence (Ota et al., 2010). Thus, the SIRT1‐eNOS‐NO axis has a protective effect against endothelial dysfunction and senescence. SIRT1 also avoids endothelial senescence by PGC‐1α and PPARα deacetylation, resulting in a decrease of both NADPH oxidase activity and NO inactivation (Zarzuelo et al., 2013).

In summary, we demonstrated that mulberry extract reduces oxidative stress in aging vasculature and attenuates endothelial dysfunction, concomitant with eNOS activity due to decreased eNOS phosphorylation at activation sites coupled with higher amino acid residue acetylation from aging. As a molecular mechanism, we have shown that mulberry extract recovered activating phosphorylation of eNOS (Ser1177) and reduced acetylation of eNOS through SIRT1 upregulation. These findings provide valuable insights into mulberry extract's effect on endothelial senescence and dysfunction in aging and beyond as other cardiovascular diseases.

## 4 EXPERIMENTAL PROCEDURES

### 4.1 Extraction and purification of Mulberry

Mulberry were collected from the South Buan Agricultural Cooperative Federation (Buan, Korea) in 2016. 70% ethanol‐soluble Mulberry was prepared and purified as previously described (G. H. Lee et al., 2019).

### 4.2 Ethics statement

All experimental animals used in this study were cared as per the protocol approved by the Institutional Animal Care and Use Committee of Jeonbuk National University Hospital (cuh‐IACUC‐2017‐9). All methods were performed in accordance with the guidelines and regulations set and approved by Jeonbuk National University Hospital.

### 4.3 Animal grouping and experimental protocol

Male Sprague‐Dawley rats were studied at 8 and 80 weeks of age. Rats were obtained from Orient Science Co (Seongnam, Korea). The rats were maintained in a 12:12‐hr light: dark cycle in a stainless‐steel wire bottom cage and acclimatized to laboratory conditions for at least 1 week before the experiments. Group 1 (*n* = 10): Young group (*n* = 10), rats were fed a normal diet and received water via oral gavage. Group 2 (*n* = 10): Young plus mulberry extract group (*n* = 10), rats received 300 mg/kg mulberry extract via oral gavage. Group 3 (*n* = 10): Aging group, rats were fed a normal diet and received water via oral gavage. Group 4, 5, 6 (*n* = 10): Aging group, rats received 100, 200 and 300 mg/kg mulberry extract via oral gavage. Experiments were terminated after 8 weeks. Rats were anesthetized with ketamine (Yuhan Yanghyang Company, Seoul, Korea), and cervical dislocation was performed.

### 4.4 Human umbilical vein endothelial cell culture

Human umbilical vein endothelial cell culture was performed as described previously (G. H. Lee et al., 2019). Cells were cultured in endothelial cell growth basal medium 2 (EBM‐2), supplemented with endothelial cell growth medium 2 (EGM‐2). After reaching 85%–90%, confluence cells were incubated with 50 mM d‐gal for 48 h. Different doses of anthocyanin and mulberry extract were added 24 h after the onset of the d‐gal challenge.

### 4.5 β‐Galactosidase staining

HUVECs at 85%–90% confluency in 6 well plates were pre‐incubated with d‐galactose (50 mM) for 48 h and then treated with different doses of mulberry extract for 24 h. The senescence phenotype was detected using the β‐galactosidase staining kit (Cell Signaling, Danvers, MA, USA). Positive senescence‐associated β‐galactosidase (SA‐β‐gal) cells were observed by light microscopy.

### 4.6 Immunoblotting

Aorta ring homogenates and cell lysates were separated on a polyacrylamide gel and transferred onto PVDF membranes. Blocked membrane was probed with primary antibodies against p‐eNOS, eNOS (Cell Signaling Technologies, Inc., Danvers, MA, USA) and p16, p21, sirtuin 1, β‐actin (Santa Cruz Biotechnologies, Inc., Santa Cruz, CA, USA). Blots were washed with TBS‐T buffer and probed again with species‐specific horseradish peroxidase‐conjugated secondary antibodies. Protein signal was visualized with enhanced chemiluminescence reagents.

### 4.7 Chemiluminescent Detection of NO Production

NO production was determined by the Griess reaction, as described previously (Lee et al., 2018). Collected supernatants were mixed with equal volumes of nitrate/nitrite assay kit (Sigma, St Louis, MO, USA), and nitrite concentration was determined as described by the manufacturer.

### 4.8 NO Imaging of Living Endothelial Cells

NO Imaging was performed as described previously (Lee et al., 2019). In short, cells were loaded with 20 µM diamino‐fluorescein‐2‐diacetate (DAF‐2DA; Sigma, St Louis, MO, USA) for 30 min at 37°C and quantified by staining with background fluorescence using epifluorescence.

### 4.9 Cellular Production of ROS Using Dihydroethidium (DHE) and MitoSOX

Intracellular ROS, including O_2_^−^, was detected as described previously (Lee et al., 2019). Washed cells and aortic rings were initially incubated with 20 µM DHE (Thermo Fisher Scientific, MA, USA) and then with 5 µM of MitoSOX (Thermo Fisher Scientific, MA, USA). Cellular areas were quantified by DHE and MitoSOX staining using background fluorescence and epifluorescence (Applied Precision Delta Vision Elite, Applied Precision Inc., Issaquah, WA, USA).

### 4.10 Dihydroxy‐rhodamine (DHR) oxidation

DHR oxidation was used to evaluate peroxynitrite production of cells exposed, as previously described (Munhoz et al., 2012). Cells and aortic ring were replaced with Dulbecco's phosphate‐buffered saline (dPBS) containing 20 µM DHR (Invitrogen, Carlsbad, CA, USA) at 37°C for 30 min. The oxidation product of DHR was detected after exposure to different experimental conditions in epifluorescence (Applied Precision Delta Vision Elite, Applied Precision Inc., Issaquah, WA, USA).

### 4.11 NADPH oxidase activity assay

The enzymatic activity of NADPH oxidase was assessed by a luminescence assay in the presence of lucigenin (250 μm) and NADPH substrate (100 μm; Sigma, St Louis, MO, USA). Fluorescence intensity was continuously monitored for 15 min with a TD20/20 luminometer. The chemiluminescent signal was corrected by protein concentration of each sample homogenate.

### 4.12 NAD^+^/NADH ratio

The ratio of NAD^+^/NADH was determined with the NAD^+^/NADH Quantification kit (BioVision, Mountain View, CA, USA). NADt (NAD^+^ + NADH) was extracted following the instructions of manufacturer. For each of the extracted samples, half of the sample was heated to 60°C for 30 min to decompose NAD^+^ while maintaining intact NADH. Both NADt and NADH samples were mixed with NAD cycling enzyme, and absorbance was measured at 450 nm using Spectra Max Microplate Luminance (Molecular Devices, Sunnyvale, CA, USA). NADt and NADH were quantified by comparing with NADH standard curve and normalizing to mg of protein. Lastly, the NAD^+^ (NADt − NADH) to NADH ratio was calculated.

### 4.13 Immunohistochemistry

Formalin‐fixed, paraffin‐embedded tissues were cut into 5‐μm‐thick sections, and immunofluorescence staining of aortic tissues was performed as previously reported. The sections were incubated overnight at 4°C with Anti‐beta Galactosidase antibody (ab9361, Abcam, Inc., Cambridge, MA, USA) diluted in Dako Antibody Diluent (S3022, Agilent Technologies, CA, USA). The sections were subsequently incubated with goat anti‐chicken IgY‐HRP (sc‐2428, Santa Cruz Biotechnologies, Inc., Santa Cruz, CA, USA) secondary antibody. Staining color was developed by Dako AEC High Sensitivity Substrate Chromogen (K3461, Agilent Technologies, CA, USA).

### 4.14 Immunofluorescence

Five‐μm‐thick sections were deparaffinized, rehydrated, and incubated in 1% BSA for 2 h at room temperature. The sections were incubated with anti‐nitrotyrosine (Abcam, Inc., Cambridge, MA, USA) and anti‐p‐eNOS (Ser1177, Cell Signaling Technologies, Inc., Danvers, MA, USA) for overnight at 4°C. Thereafter, the sections were labeled using anti‐mouse IgG‐TRITC and anti‐rabbit FITC (Sigma, St Louis, MO, USA). Finally, the samples were counterstained with DAPI, mounted, and captured using EVOS M5000 Cell Imaging System (Thermo Fisher Scientific, MA, USA).

### 4.15 Protein acetylation measurements

Immunoprecipitation was used to selectively isolate eNOS and quantify its acetylation status. Treated cells were lysed, total protein extracted, and protein content measured in the supernatant by the Bradford method. A total of 0.3 mg protein was precleared by adding 1 µg of normal rabbit IgG control and 20 µL protein agarose and mixed for 1 h (4°C) with a subsequent centrifugation at 12,000 *g* for 10 min at 4°C. The supernatant was recovered and incubated at 4°C, subjecting to mild agitation overnight with 10 µl of an eNOS immunoprecipitation antibody. Twenty μl of protein A/G‐Sepharose was added, and the mixture was incubated overnight at 4°C with shaking. The immunoprecipitation mixture was centrifuged at 12,000 *g* for 15 min at 4°C, and the supernatant was recovered and stored at 4°C for later analysis. The pellet was washed 2×Sample Buffer, shaking for 15 min, and centrifuged at 12,000 *g* for 15 min at 4°C. The immunoprecipitation proteins in the pellet and those remaining in the supernatant were applied to a precast 4%–15% sodium dodecyl sulfate–polyacrylamide gradient gel electrophoresis for Western blotting against anti‐SIRT1 (Santa Cruz Biotechnologies, Inc., Santa Cruz, CA, USA) and anti‐acetylated lysine (Cell Signaling Technologies, Inc., Danvers, MA, USA) for immunoblotting respectively.

### 4.16 Statistical analysis

All statistical analyses were performed using GraphPad Prism version 8.0 (GraphPad Software, San Diego, CA, USA) software. Data were expressed as mean ± standard error of the mean (SEM). The groups were compared using one‐way ANOVA with Tukey post hoc comparison, and *p* < 0.05 was considered as statistically significant.

## CONFLICT OF INTEREST

The authors declare no conflict of interest.

## AUTHOR CONTRIBUTIONS

G.‐H.L. and T.‐H.H. conducted the experiments and analyzed the data. S.‐J.J. and E.‐S.J. partially performed some experiments, and S.‐W.C. and H.‐J.C. supervised and participated in the data analysis and interpretation. All authors reviewed the manuscript.

## Supporting information

Fig S1

Fig S2

Fig S3

Supplementary Material

## Data Availability

Data sharing not applicable to this article as no datasets were generated or analyzed during the current study.
