# Supplementary figures and images for: Anthocyanins attenuate endothelial dysfunction through regulation of uncoupling of nitric oxide synthase in aged rats
Source: Aging Cell. 2020 Dec 3;19(12):e13279. doi: 10.1111/acel.13279 (PMC7744959; doi:10.1111/acel.13279)

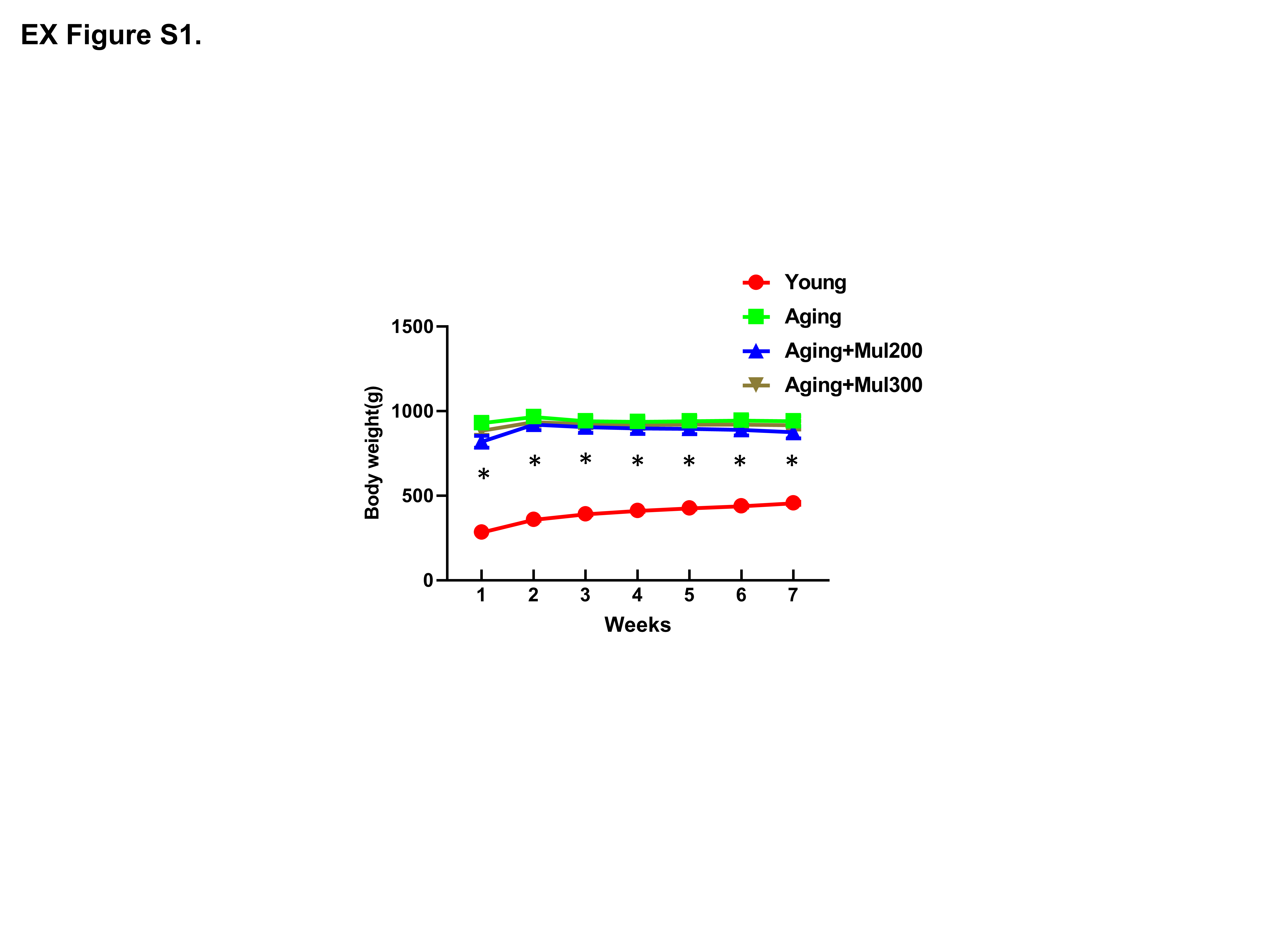

Supplement: Supplementary file 1 — Fig S1 [file ACEL-19-e13279-s001.TIF]

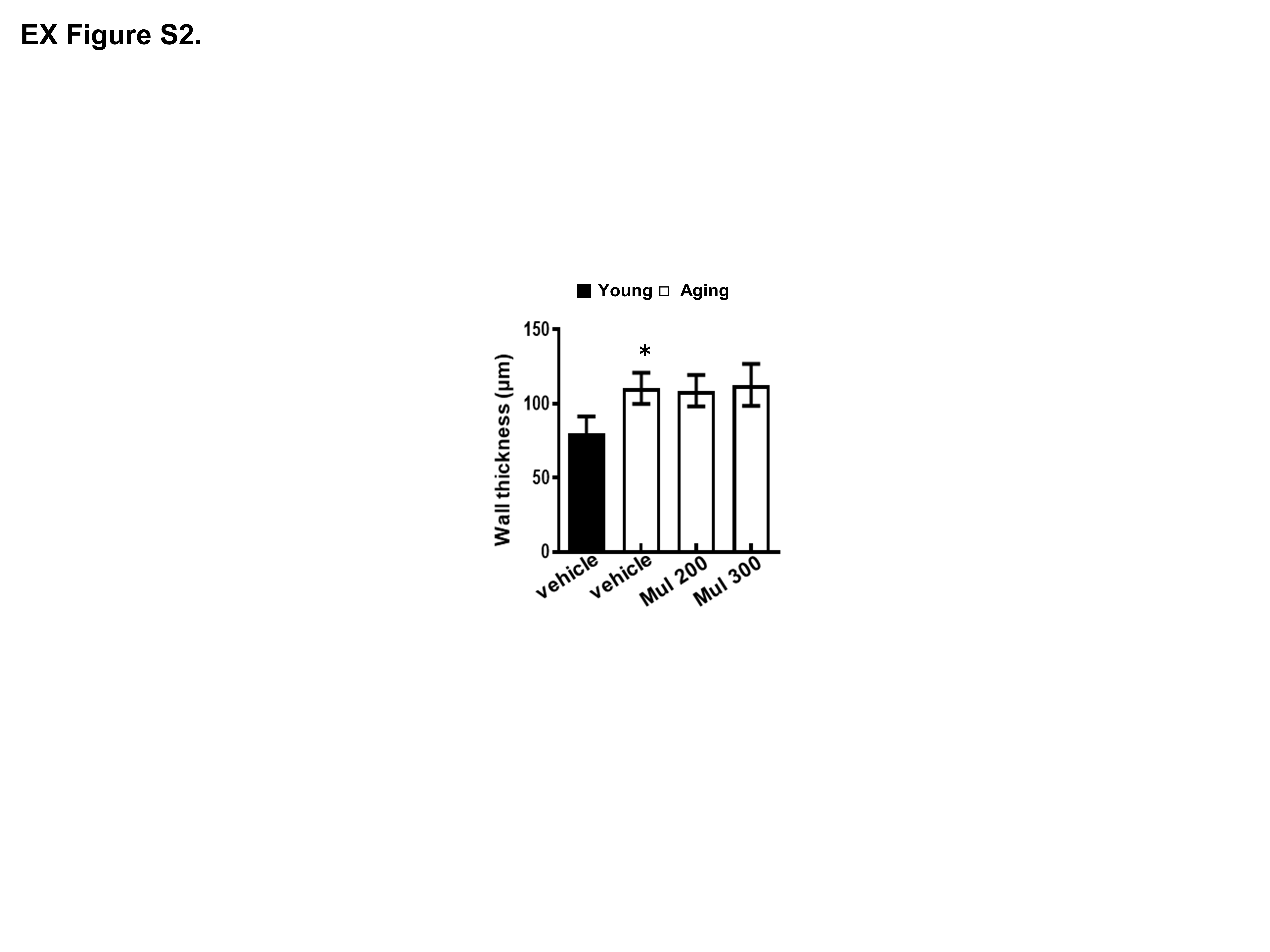

Supplement: Supplementary file 2 — Fig S2 [file ACEL-19-e13279-s002.TIF]

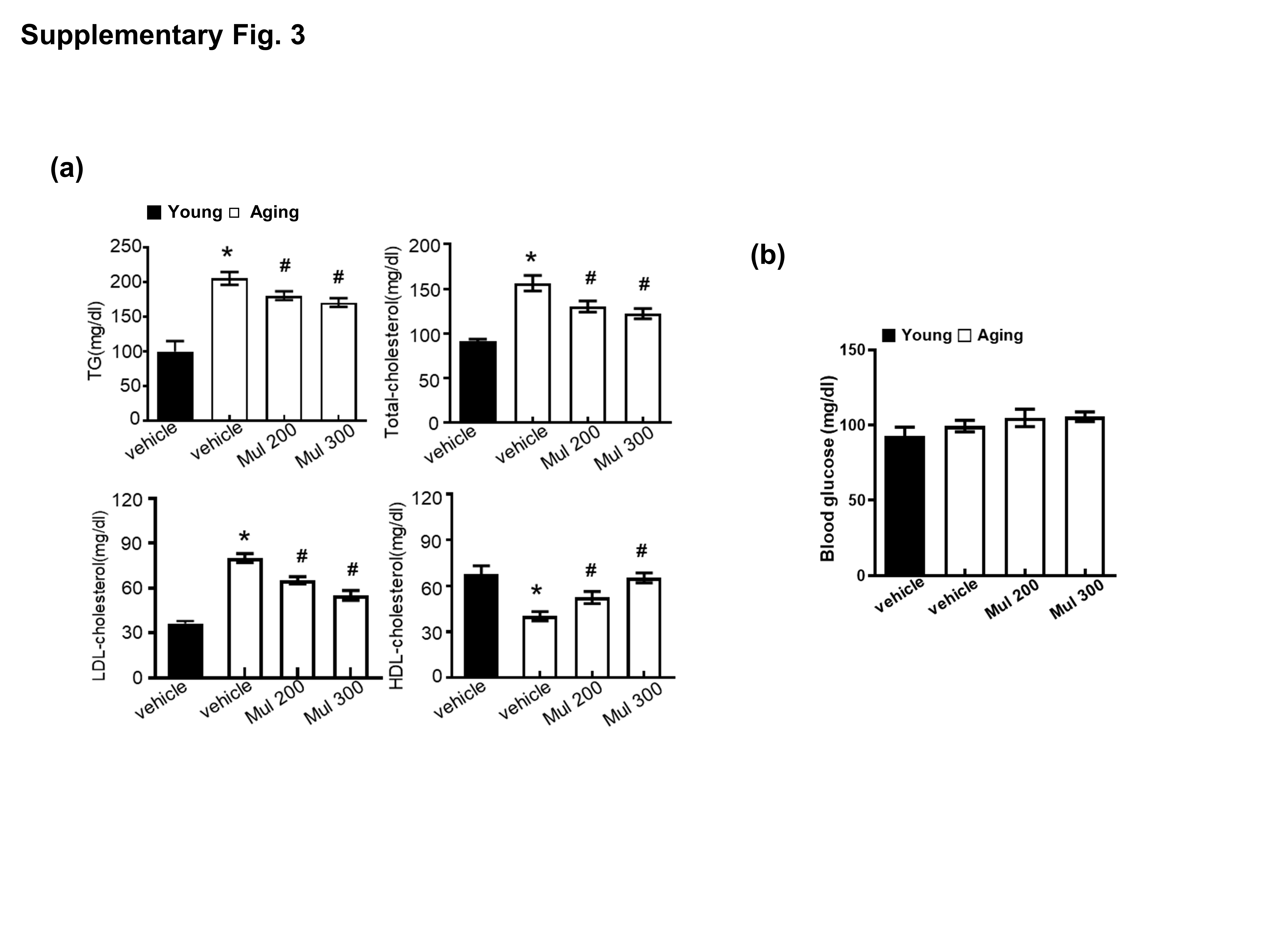

Supplement: Supplementary file 3 — Fig S3 [file ACEL-19-e13279-s003.TIF]
