# Supplementary material for: Anthocyanins attenuate endothelial dysfunction through regulation of uncoupling of nitric oxide synthase in aged rats
Source: Aging Cell. 2020 Dec 3;19(12):e13279. doi: 10.1111/acel.13279 (PMC7744959; doi:10.1111/acel.13279)
Supplement: Supplementary file 4 — Supplementary Material [file ACEL-19-e13279-s004.docx]

**Extended figures**

**Ex Figure S1**

Mulberry supplement effect on body weight in aging rats. ^*^*P* < 0.05 versus Young groups. Data are presented as mean ± SEM.

**Ex Figure S2**

Mulberry supplement effect on wall thickness in aging rats. ^*^*P* < 0.05 versus Young groups. Data are presented as mean ± SEM.

**Ex Figure S3**

Effects of mulberry on the lipid profile (**a**) and Fasting blood glucose level (**b**) of aging rats after eight-week long treatment. HDL-C, HDL-cholesterol; LDL-C, LDL-cholesterol; TC, total cholesterol; TG, triglyceride. ^*^*P* < 0.05 versus Young groups. ^#^*P* < 0.05 versus Aging groups. Data are presented as mean ± SEM.
